# Tracing the undercurrents: a scoping review of the lifestyle drift concept

**DOI:** 10.1186/s12889-025-25616-6

**Published:** 2025-12-23

**Authors:** Tanya Halsall, Heather Orpana, Misha Jan

**Affiliations:** 1 University of Ottawa Institute of Mental Health Research at the Royal, Ottawa, Canada; 2 Department of Neuroscience, Carleton University, Ottawa, Canada; 3 School of Epidemiology and Public Health, University of Ottawa, Ottawa, Canada; 4 Centre for Surveillance and Applied Research, Public Health Agency of Canada, Ottawa, Canada; 5 School of Psychology, University of Ottawa, Ottawa, Canada; 6 Department of Psychology, Carleton University, Ottawa, Canada

**Keywords:** Public health, Lifestyle drift, Health equity, Interventions, Upstream prevention, Neoliberalism, Biomedical model

## Abstract

**Background:**

Lifestyle drift refers to the tendency for health interventions that are initially intended to address upstream social determinants of health to shift focus toward downstream efforts targeting individual behaviours. Although increasingly cited in the literature, as far as we are aware, no scoping or systematic reviews that examine the concept of lifestyle drift exist. The objective of this scoping review is to summarize the existing literature on lifestyle drift, describe how it is defined in the literature, the causes or mechanisms of influence that lead to lifestyle drift and ways to address it.

**Methods:**

A comprehensive search strategy was developed with guidance from a librarian and seven databases were searched. Title and abstract and full text screening was conducted in Covidence according to inclusion and exclusion criteria. Included documents were imported into NVivo, and data from articles were analysed using a thematic analysis approach.

**Results:**

We identified 318 articles and 32 met the inclusion criteria with lifestyle drift as a focus or major theme. Definitions of lifestyle drift shared some common elements along with variability of concepts. Neoliberalism and the biomedical model were frequently identified as drivers of lifestyle drift across articles. While many strategies to counteract lifestyle drift were proposed, applying a health in all policies approach, and participation from priority populations were the most common strategies suggested.

**Conclusion:**

While lifestyle drift is a recognized concern in public health, health promotion and chronic disease prevention, there remains a need for more empirical research on lifestyle drift, including mechanisms and mitigation. Strengthening understanding of how to identify, prevent, and address lifestyle drift may enhance the effectiveness of upstream interventions aimed at reducing health inequities.

**Supplementary Information:**

The online version contains supplementary material available at 10.1186/s12889-025-25616-6.

“Merely to do more of what we have always done is not an option. A paradigm shift in thinking is needed.” (Hunter, 2010, p.1)

## Background

Lifestyle drift has been identified as a prevalent issue in public health, characterized by a tendency to acknowledge the importance of acting on the ‘upstream’ structural determinants of health, only to divert attention ‘downstream’ to implement interventions with an emphasis on individual behavioural determinants of health [1]. Scholars have proposed possible causes of lifestyle drift, for example perceived feasibility and cognitive accessibility of interventions focussed on the individual, a greater level of evidence on behavioural interventions, and a culture of personal ‘responsibilization’ for health [1]. Given public health’s goal to improve overall health and reduce inequities, and the emphasis placed on action targeted toward social determinants through upstream interventions, it is important to identify, understand, measure and monitor lifestyle drift. By doing so, we can bring awareness to a phenomenon that may hamper initiatives aimed at enhancing overall population health and reducing health inequities.

To our knowledge, there are no existing narrative, scoping or systematic reviews focussed on detailing how the concept of lifestyle drift is described in the literature. Scoping reviews are a method of evidence synthesis that aim to systematically document evidence, and can be used to “identify, map, report or discuss the characteristics or concepts in a field” [2] p. 2121. Strengths of the scoping review methodology include the systematic approach that is applied, transparency, flexibility, and the ability to synthesise a wide range of evidence sources. Generally, scoping reviews adhere to the following steps: identification of the research question, inclusion criteria, concept, context, and participants (where applicable); types of evidence sources; development of a search strategy; evidence screening and selection; data charting; analysis of evidence; and presentation [2].

In order to address the above gap, we are conducting a scoping review of published literature on this concept. The objective of this scoping review is to characterise how lifestyle drift is defined in the literature, what factors contribute to the occurrence of lifestyle drift and an examination of how it can be mitigated. This information contributes to an overall purpose of developing a better understanding of lifestyle drift to use this information to enhance the implementation of upstream prevention strategies.

## Methods

This scoping review was conducted following the framework described by Arksey and O’Malley [3] and further expanded on by Peters et al. [2]. The protocol for this scoping review was published on the Open Science Framework at before data charting commenced [4].

Within this scoping review, the realm for the data collection is published literature that includes reference to the concept of lifestyle drift in the domain of public health or health. The following databases were searched: Scopus, Embase, Medline, PsychINFO, ProQuest, and OVID Global Health and CAB. Google Scholar was also searched and the first 10 pages of articles were included in screening, consistent with the approach adopted by Godin et al. [5]. This approach balances comprehensiveness with feasibility. Three seminal articles from the literature on lifestyle drift were also searched using “Connected Papers” [6] and related articles were included in screening. The search terms used are documented in Appendix A. The search covered literature published up to March 2023, ensuring the review’s relevance to current discussions on lifestyle drift.

Retrieved articles were uploaded into Covidence, an online platform for conducting systematic and scoping reviews, and deduplicated [7]. Title and abstract screening was conducted independently by two reviewers (from among HO, MJ, or TH). Full text review was conducted by one reviewer. Reasons for exclusion at full text review were documented.

**Table 1:** Inclusion and exclusion criteria

Domain	Inclusion criteria	Exclusion criteria
Content	Document must explicitly reference the concept of lifestyle drift within the body of the document, not only in the references	Document does not explicitly reference the concept of lifestyle drift or lifestyle drift is only found in the reference list
Language	English language	Not in English
Document type	Research papers, commentaries, editorials, conceptual papers, literature reviews	Books, book chapters, letters, conference abstracts, grey literature
Discipline	Publications within the disciplines of health and public health	Publications outside of the disciplines of health and public health

During full text review we identified that a large number of articles met the original inclusion criteria. In order to keep the body of literature being reviewed manageable, we applied an additional set of inclusion criteria by sorting documents that referenced the concept of lifestyle drift into two groups, and only including documents where lifestyle drift was either the focus of the document or a major theme, based on the criteria of “lifestyle drift appears at least two or more times in document or in the title or significant discussion in three or more paragraphs about LD in document.” We excluded documents where lifestyle drift was a minor focus or a passing reference, based on the criteria of “lifestyle drift appears once or twice in document and discussion of LD is limited to one or two paragraphs.”

Aligned with study objectives, an initial code book was created for the categories described in Appendix B. As described in the original protocol [4] we intended to chart data according to Rodgers’ evolutionary concept analysis [8, 9]. However, after piloting this approach we found that the framework contributed to many overlapping codes that did not contribute to a more meaningful depiction of the concept. Therefore, we determined that a more inductive approach would allow for a better understanding of the concept. To this end, we applied thematic analysis to allow coding categories to be generated through familiarization with the data [10, 11]. This included becoming familiar with the data, generating initial codes, searching for themes, reviewing and refining themes, defining and naming themes, and writing the report [10]. To ensure the coherence of the coding process, a pilot test was conducted on a subset of three studies initially with two reviewers. The reviewers met to compare their codes, discuss discrepancies, and adapt initial codes accordingly. For the full dataset, three coders analysed a subset of articles. A second coding of the full dataset was completed by TH. The codes were then reviewed by HO and TH to come to consensus. Bibliographic information, type of document, discipline of first author based on institutional affiliation, geography of either the study or based on the location of the first author, and year of publication were also recorded for analysis.

Critical appraisal of published data sources was not conducted as this was not necessary to meet the scoping reviews goals of characterising how lifestyle drift is defined in the literature, what factors may contribute to the occurrence of lifestyle drift and an examination of how it can be mitigated. We applied the PRISMA-ScR checklist extension for scoping reviews [12], which can be found in Appendix C.

## Results

**Fig. 1:**
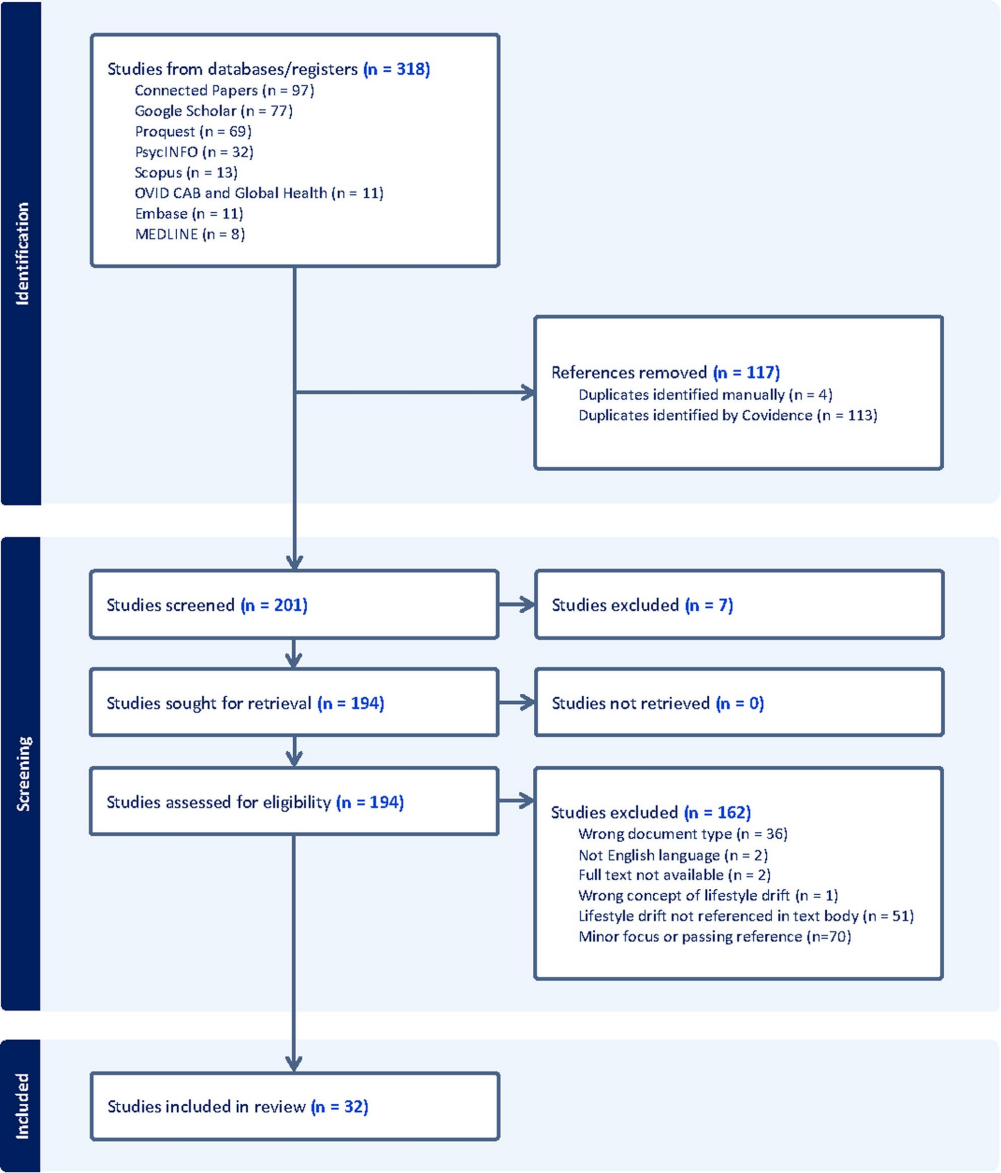
PRISMA diagram

As shown in the PRISMA diagram in Fig. 1, our searches identified a total of 318 articles, 117 of which were duplicates. A total of 201 articles were screened at title/abstract screening, with 194 articles moving to full text screening. At full text screening, 162 studies were excluded, with 70 of these being excluded because lifestyle drift was only a passing reference or minor focus of the document.

A total of 32 studies were included in the final review. Most articles were authored by a first author citing an affiliation located in Australia (*n* = 10), followed by the United Kingdom (*n* = 9), Canada and England each at (*n* = 4), the Netherlands (*n* = 2), Scotland (*n* = 1) and Spain (*n* = 1). There is a noted lack of articles led by authors in the United States, Asia, Africa or South America.

**Fig. 2:**
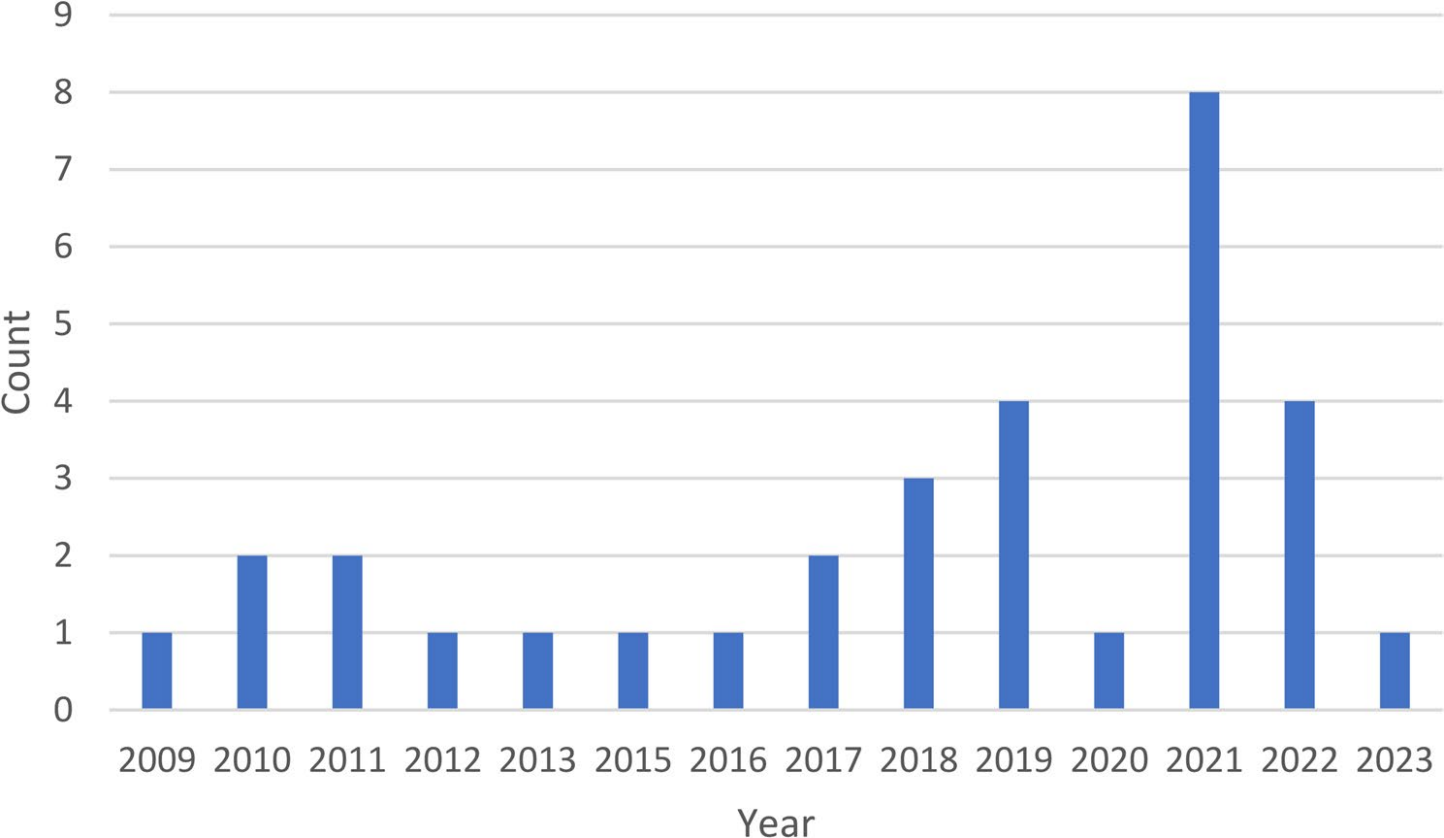
Number of articles included in scoping review by year of publication

As shown in Fig. 2, there was an increasing number of articles included in our review with later year of publication, likely indicating a spreading awareness and interest in the concept of lifestyle drift. Indeed, in 2021, there were twice as many articles that were included in our review as from any previous year.

Table 2 presents articles, year of publication, discipline of first author, country of first author or article focus, and recommendations from articles on how to address lifestyle drift.

**Table 2:** Article characteristics and summary of recommendations from articles to address lifestyle drift

Article	Year	Discipline (of first author)	Country (of article focus or first author)	Summary of Recommendations
Baum, 2011 [13]	2011	Health, Society and Equity	Australia	Apply lessons learned from successful case examples and analyze previous work on SDoH (Social Determinants of Health)
Baum et al., 2019 [14]	2019	Health, Society and Equity	Australia	Create legislative mandate focused on HiAP; partner with citizen groups; evaluate health in all policy efforts through partnership with academics (also capture barriers and facilitators)
Baum et al., 2018 [15]	2018	Health, Society and Equity	Australia	Form relationships with policy makers; build policy-related evidence; monitor fidelity to SDoH, policy silences and successful case examples
Berg et al., 2021 [16]	2011	Public health	Netherlands	Balance individual and collective responsibility for health; give voice to disadvantaged populations
Bournival et al., 2022 [17]	2022	Medicine	Canada	Target SDoH that create vulnerabilities to support disaster response; shift focus from preparedness to prevention; resist quick-fix and siloed strategies
Brookes, 2021 [18]	2021	Linguistics	United Kingdom	Balance public health discourses to highlight lifestyle factors along with social determinants
Capper et al., 2023 [19]	2023	Public Health	England	Shift policy focus to SDoH; collaborate with individuals experiencing health inequalities; employ proportional universalism; transfer power and resources with responsibility to reduce health inequalities
Carey et al., 2017 [20]	2017	Public Administration Public Health	Australia	Reframe obesity as population issue through highlighting systematic differences; frame the problem of obesity as influenced by environment so that government will accept responsibility; frame as human rights issue to protect children; frame integration of universalism and targeting
Collins et al., 2015 [21]	2015	Media, Culture and Society	Scotland	Bridge the gap between evidence on macro-level processes and policy development and implementation
Dawson et al., 2021 [22]	2021	Psychology	Australia	Apply strengths-based interventions that go beyond an individual level
Godziewski, 2021 [23]	2021	Sociology and Policy	Europe	Adopt a wellbeing economy
Green et al., 2022 [24]	2022	Disability and Health	Australia	Develop policy solutions for populations with disability that consider micro, meso and macro level factors; apply theory and research evidence to inform policy advocacy; "break down" actions to address SDoH so that components can be addressed within individual government departments but ensure that they are also coordinated; consult with individuals with lived experience of disability; engage policy entrepreneurs; policy advocates strengthen awareness of policy processes and structures
Hunter et al., 2009 [25]	2009	Health Policy and Management	England	Increase local ownership and highlight inter-relationships among SDoH; create boundaries to the operations of the market; increase social supports that will enhance social cohesion and cooperation; support redistribution through taxes, wherein wealthy receive benefits of a more cohesive society; prioritize social development in policy; apply transformational leadership approaches; co-create knowledge and co-produce solutions; frame issues from policy, rather than practice; encourage risk-taking and ability to be comfortable with uncertainty; harness champions; visualize whole systems and apply complex systems thinking and quality improvement; attend to social gradient rather than priority populations
Hunter et al., 2010 [26]	2010	Health Policy and Management	United Kingdom	New kinds of partnership, e.g. “whole area” approaches; increase investment in the early years and ensure progressive distribution across social gradient; use skills building to reduce the gradient; create quality employment opportunities for all; progressive taxation and related policies; create healthy communities; invest in all government departments to reduce the social gradient; flexible leadership; community empowerment; support “shift in culture” and practice change through an organic approach
Johnson & Woodall, 2022 [27]	2022	Health	United Kingdom	Apply place-based strategies
Kirkland & Raphael, 2018 [28]	2018	Health Policy and Management	Canada	Raise awareness about the need for a focus on SDoH
McGowan et al., 2021 [29]	2021	Population Health Sciences	United Kingdom	Reduce the social gradient through proportionate universalism; apply universal interventions that require less agency to achieve health benefits; apply more economic interventions to reinforce physical activity
MacKay, 2021 [30]	2021	Health Ethics	Australia	Address maternal needs through policy change; create policy to support healthy childhood nutrition
Perello, 2020 [31]	2020	No affiliation	Spain	Pursue structural reform and collective action; limit the free market and redistribute wealth
Phillips et al., 2016 [32]	2016	Health, Society, and Equity	Australia	Broaden strategies to other government departments outside of health
Popay et al., 2010 [33]	2010	Sociology and Public Health	England	Develop more flexible leadership and partnership approaches focused on place-based strategies; resist short-termism; build social movement; raise awareness; empower communities
Powell et al., 2017 [34]	2017	Public Health	United Kingdom	Move away from short-termism and less restricted monitoring approaches
Rich et al., 2019 [35]	2019	Health	England	Harness digital innovation to support health policy and collective response; have policymakers engage with the contexts that influence access and engagement with health-related technologies
Raphael et al., 2019 [36]	2019	Health Policy and Management	Canada	Raise awareness of commercial determinants of health and evidence regarding social determinants if health; empower communities
Roesler et al., 2021 [37]	2021	Health, Society, Equity	Australia	Need for federal and state leadership to support local strategies; need for a coordinating structure to monitor and inform regional activities
Schrecker, 2013 [38]	2013	Epidemiology and Community Medicine	Canada	Need for a focus on macro-level strategies; raise awareness with health promoters and revise strategic focus
Shakespeare et al., 2021 [39]	2021	Health, Society, Equity	Australia	Apply an Indigenous relational perspective to inform policy development
van Baar et al., 2023 [40]	2023	Mental Health and Addiction	Netherlands	No recommendations as lifestyle drift was not prevalent
Watson et al., 2021 [41]	2021	Health and Society	United Kingdom	Recognize inequities in autonomy for food choices and complexity in decision-making regarding nutrition; require more accountability of food industry
Watt & Sheiham, 2012 [42]	2012	Epidemiology and Public Health	United Kingdom	Distribute health services relative to need; apply culturally-informed approaches; coordinate national local-level policy; focus policy on supporting oral health, with special consideration for the early years; intersectoral partnership; empower communities
Williams & Gibson, 2018 [43]	2018	Health	United Kingdom	Include qualitative research that accounts for social context; apply social intervention to increase equitable access; follow Behavioural Justice agenda; conduct more interdisciplinary research on physical activity behaviour
Williams & Fullagar, 2019 [44]	2019	Health	United Kingdom	Recommend a more in-depth analysis of past social and political influences on service provision to identify opportunities for improvement

### Insight regarding the definition

The overall purpose of the scoping review was to develop a better understanding of how lifestyle drift is defined in the literature, the mechanisms of influence that lead to lifestyle drift and ways to address them. Our main themes are focused on: the ways that lifestyle drift are defined, proposed causes and proposed mitigation strategies.

**Fig. 3:**
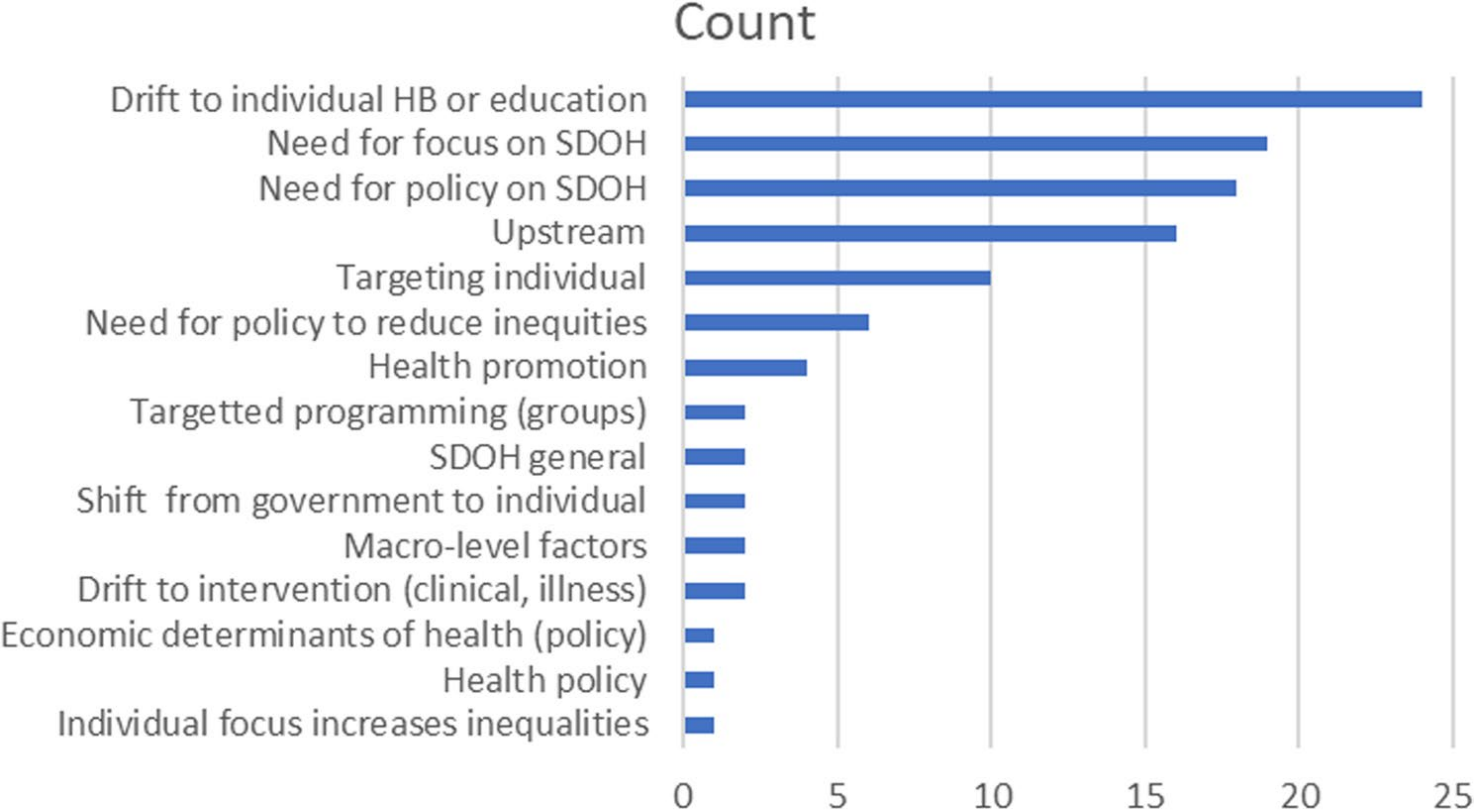
Relative frequency of concepts and terms in lifestyle drift definitions

One of the most common definitions cited in the literature comes from Popay, Whitehead and Hunter [33], “‘lifestyle drift’—the tendency for policy to start off recognizing the need for action on upstream social determinants of health inequalities only to drift downstream to focus largely on individual lifestyle factors. Coupled with this is a move away from action to address the social gradient towards activities targeted at the most disadvantaged.” (p. 148). While coined in the context of policy, this concept may apply beyond policy to other intervention formats. In order to capture a better understanding of how lifestyle drift is defined in the literature, we analyzed the specific terminology and concepts that are being included in the definitions provided within the studies. Twenty-four of the 32 articles we reviewed included a specific definition of lifestyle drift. Nearly all definitions contained some description of a drift toward either health behaviours or lifestyle factors (*n* = 24) and an acknowledgement of the need to focus on structural or social determinants of health (*n* = 19). Many also included the term upstream (*n* = 16), some mention of targeting individuals (*n* = 10) and policy on the social determinants of health (*n* = 8). Figure 3 displays the relative frequency of the language used.

It is interesting to note that although most concepts and issues are overlapping across definitions, there are two criteria that are included in only a small subset of definitions, yet, as we will discuss below, there are many instances of these processes described within the literature more broadly: (1) Drift to targeted programming for priority populations [25, 33] and (2) Drift to treating illness [15, 32]. We re-visit the phenomenon of drifting to treating illness again within the below section on the biomedical model, but provide further elaboration on drift to targeted programming here.

Within our included studies, there was a sub-group that described a process whereby lifestyle drift is perpetuated by initiatives that apply strategies that are targeted to a small sub-group of the population. These are often priority populations or individuals at higher risk with respect to a certain health outcome [20, 24, 33]. Baum and Fisher [45] describe this issue in further detail

Contemporary behavioural-health promotion strategies fall into two broad types; those applied across a large population (universal), and those implemented in a local area or in an identified at-risk group (targeted)… Small-scale targeted strategies such as intensive behaviour-change interventions with high-risk individuals have produced some limited positive results (for example, Laatikainen et al. 2007). These trials require significant resources and may produce benefits for a small group, usually those with other aspects of their life are going well. However, this form of evidence is not helpful in terms of changing risk factors across a whole population because even a large change in such a small proportion of the population will not have any significant effect on overall population health (Chapman 1985, Rose 1992), and the intensive intervention methods required are not feasible on a large scale. (p. 215)

A shift to targeted programming appears to be a well-documented occurrence across prevention initiatives, despite its relative absence from conceptual definitions of lifestyle drift.

### Proposed causes: neoliberalism

Recognizing that the factors that contribute to lifestyle drift are complex and perpetrate influence at the system-level, many of the factors are overlapping. Yet, highlighting and defining them can support researchers, practitioners and policy-makers to better recognize how they exert their influence and hopefully also support them in recognizing them within their own work.

There were two main themes identified that were most commonly noted across publications: neoliberalism (*n* = 16) and the biomedical model (*n* = 15). Neoliberalism represents

“the doctrine that markets are the normal, natural and preferable way of organizing most forms of human interaction; that any departure from markets and the allocation of resources they generate requires justification to a high standard of proof; and that the primary function of the state is to ensure the functioning of markets, even when this requires intrusive or coercive measures” [38].

The biomedical model represents a scientific paradigm that explains illness as a physical dysfunction that manifests at a lower-level of the organism [46]. This perspective aligns with Cartesian dualism and reductionist approaches [46, 47] that apply linear and causal logic and convergent solutions that are not well-suited to complex social systems [25, 48, 49]. The biomedical model has remained dominant over other paradigms within health systems [22, 24, 32, 39, 40] in spite of substantial levels of ill health in the population broadly and persisting and profound health inequities [49]. Although these two factors are intertwined in their contribution to lifestyle drift and frequently appear in combination within government and institutional approaches [36, 37], they are often defined separately in the literature and reflect largely distinct mechanisms of influence.

We further subdivided the neoliberalism theme into four sub-themes that described phenomena that were all influenced by neoliberal ideology and closely inter-related yet maintained distinguishable characteristics. The first subtheme addresses the belief that individuals will change behaviour based on knowledge, placing responsibility on the individual. The subsequent three are economic drivers, commercial determinants of health and political processes. The biomedical model theme contains one subtheme: preference to focus on healthcare or clinical interventions.

Researchers identified neoliberal ideology (along with related concepts including individualism, capitalism, consumerism, liberal welfare state and advanced liberalism) as being a central driver behind lifestyle drift as well as many of the principles and processes that play a significant role [13, 28, 33, 36, 44]. Many researchers identified that neoliberalism had become a dominant influence across Canada, Australia, the UK as well as other European countries [15, 16, 34, 36, 44]. Notably, only two studies included researchers from the United States [27, 40] and neoliberalism was not discussed in these articles. Researchers described the role that neoliberalism plays in supporting government shifts toward a focus on supporting market competition, increasing privatization and decreasing regulation to promote economic growth along with a concurrent retreat from the provision of social supports [15, 18, 25, 44]. This orientation is combined with the notion that individuals must take responsibility for their own health and that this role is implicit in their civic participation [15, 16, 18, 32, 34, 35, 41]. This notion has been instrumental in the pervasive emergence of lifestyle drift across health promotion spheres.

Neoliberalism operates in concert with the biomedical model (see below) to reinforce a focus on the individual as both approaches neglect to take environmental influences into account and prioritize the application of behaviourally focussed interventions [24, 37, 38]. We will discuss how this translates into interventions focused on health behaviours within the following sub-theme.

Integral to the concept of lifestyle drift is the practice of placing responsibility on the individual to change their behaviour and a belief that the main problem is that individuals are lacking sufficient health-related knowledge. Researchers have remarked that this approach presents an intuitive appeal [19]. As noted by Baum [13]:

there is an inherent logic to the intention of social marketing campaigns. The idea of people wanting to live longer and healthier and so responding to lifestyle messages and adopting smoke-free, moderate alcohol, low-fat and sugar, and active lifestyles has a ring of truth to it.

This principle and practice is, in part, based on the notion that individual behaviour is guided by rational logic, whereby individual health behaviour is conceived to be driven by rational choices that are based on information [41]. Further, researchers maintain that this position is embedded within neoliberal perspectives as demonstrated by the orientation of the *Tackling Obesity* initiative:

Another aspect of the neoliberal discourses espoused by the *Tackling Obesity* policy paper is that it positions the public as informed and “rational” citizen-consumers who, if they can only harness more information and knowledge with respect to obesity and risk, will then act in the interests of their health and for the good of the state and its health care system [18].

These beliefs underpin efforts focused on changing individual behaviour rather than examining how the environment can be transformed to support health [15]. Using a case example of a community initiative to promote physical activity, researchers identified that this shift in responsibility, and therefore blame, can apply to both individual behaviour change, but also practitioner responsibility and that these strategies persist, regardless of the need to recognize that individuals may not have the means to take ownership [44]:

Shifting responsibility, and ultimately individualising it, is of course an implicit aim of advanced liberal governance…. (p. 29) Almost inevitably a culture of blame shifting emerges as fingers are pointed at individuals, whether that be the manager of a service provider, or a sedentary resident…. Rather than offering adequate support, the initial subsidy was a strategy to ‘enlighten’ a low-SES population to support them to make ‘better choices’…While there may be some legitimacy to this argument in relation to more affluent populations, in this context it undermines the aim of promoting lifestyle modification in a severely deprived neighbourhood. (p. 32)

As a central component of neoliberalism, the role of the economy was outlined by a large number of authors [14, 24, 33, 35, 38, 42–44]. Economic factors were closely intertwined with influence from industry as well as political ideologies that prioritize the market, however, the economy, itself, was described as playing a unique role. Economic growth is often prioritized over health and equity:

Public policy in general and welfare systems in particular mimic markets in the search for economic efficiency and higher productivity. Social life and relationships—the ethics of care—are secondary, and must adapt, to the work ethic and economic growth [33].

As such, poor economic conditions diminished efforts focused on health in all policy initiatives [14] and influenced preferences to invest in low-cost health solutions [35].

Another factor that is closely related to neoliberalism and lifestyle drift is the commercial determinants of health. Researchers identified that lifestyle-focused interventions were a strategy that is often endorsed by the corporate sector [28, 36] and that these strategies have been used to align industry with healthcare [43]. Corporate involvement on institutional boards can also serve to maintain strategic focus on lifestyle factors [28, 36]. According to Godziewski [23], industry can exert disproportional influence in policy and program development by leveraging their access to significant resources and representing larger networks of corporate members. Finally, case examples were identified where governments avoided associating negative health impacts with industry influence:

… When [factors outside of individual choice] are hinted at, such as the role of food and drink manufacturers and marketers, the precise roles of these organizations in contributing to obesity are backgrounded or mitigated, being more likely to be presented positively on the rare occasions that they [are] mentioned explicitly [18].

Political processes beyond the neoliberal ideological frame were also highlighted within a range of publications. For example Philips and colleagues [32] noted that policy movement on health equity issues is undermined by a variety of factors, including the high frequency of election cycles and the need for longer timelines, ineffective knowledge exchange between policy makers and researchers, challenges with intersectoral collaboration, lack of advocacy for social determinants of health, lack of consensus on effective solutions, influences from the medical profession and preference to invest in healthcare.

Godziewski [23] stresses that policy solutions that maintain the status quo receive preference and that policymakers are less receptive to research evidence that is not reported in “normatively neutral” language. Finally, challenges arise as efforts focused on social determinants of health must span government departments [24, 38], while there is a preference to address issues that can be situated easily within one department [24]. Further, key actors are often situated within government structures “that are subject to strict political direction and control, and have little direct influence on broader social determinants of health” [38].

### Proposed causes: biomedical model

The biomedical model featured as a key issue across many of the included papers [22–25, 28, 32, 36, 37, 39, 43]. Schrecker [38] describes how medical disciplines and what they privilege as evidence, supports the dismissal of recommendations to promote health equity:

[The contrast between Canadian individualism in health promotion and the] WHO recommendations: to “tackle the inequitable distribution of power, money, and resources”… may also reflect protagonists’ training in such disciplines as medicine, nursing or epidemiology, which are (with rare exceptions) relentlessly focused on the individual patient or client, and on micro-level interventions that address just one aspect of daily life, with the randomized controlled trial as the gold standard. (p. 54–55)

This medicalization was recognized as affecting efforts focused on reducing obesity [23], people with disabilities [24], Indigenous peoples [22, 39], pain management [27], men’s health [28], children and youth [32], heart disease [36], and the general population [37, 38, 40, 43, 44].

The critical influence of the biomedical model is translated into a near singular focus on the healthcare system and dismissal of the potential of health promotion opportunities within other contexts [15, 24, 39]. This practice also fails to recognize Indigenous conceptions of wellbeing or to address the holistic needs of Indigenous communities [39]. Similarly, the biomedical model perceives individuals with disabilities from a deficit lens and serves to intensify focus on health services, rather than structural determinants [24]. Several researchers drew attention to the fact that the health promotion policy was often primarily focused on health care or clinical populations [32, 37]. This is exemplified even by intersectoral efforts that are intended to create more comprehensive impacts on health, but often replicate medical and behavioural approaches that do not influence social determinants of health [15].

### Proposed mitigation strategies

Among a range of proposed mitigation strategies, the following were more commonly recommended: (1) support participation from priority populations, (2) apply health in all policies approaches, (3) apply proportional universalism, (4) apply flexible leadership approaches, (5) draw lessons learned from successful case examples, (6) apply place-based local approaches, (7) coordinate national/federal and provincial/state support with local initiatives, (8) invest in the early years (9) support intersectoral partnership and (10) raise awareness about evidence for strategies focused on the social determinants of health. We have itemized the recommendations presented by the authors of the literature reviewed and presented them in Table 2. Of note, there were also many recommendations provided by researchers regarding addressing gaps and for future research studies that could also contribute to supporting an increased focus on social determinants, but we included them in a separate category and these are not included in this manuscript.

The two recommendations that were most commonly presented were to support inclusion of priority populations and to apply health in all policies approaches. Health in all policies was mentioned in 8 publications as a viable way of moving forward in addressing the social determinants of health. This approach has been described as:

a public policy agenda that … aims to mainstream health equity, protection and promotion, across policy areas… Rather than being a policy, [health in all policy] represents a ‘way of working’, a policy agenda which embodies a normative vision for a society in which wellbeing and social justice is a central objective [23].

Finally, supporting participation of priority populations was identified as a recommendation within ten of the publications. These strategies ranged from including the voices of priority populations in governance [36], collaboration with patients and co-creation with children and youth [35], community empowerment [19, 33, 42], consultation with people with disabilities [24], partnership with Indigenous communities [22], partnership with citizen groups [14], giving disadvantaged citizens a voice [16] and building trust with residents [34].

## Discussion

This scoping review was designed to examine the literature on lifestyle drift in order to analyse how it has been defined, explore the contributing factors and review the recommendations to mitigate lifestyle drift. The information presented in this scoping review can be used by those developing programs and policies to first of all, be aware that lifestyle drift exists; to identify some of the factors driving lifestyle drift; and with this knowledge, actively work to counteract lifestyle drift.

One of our key observations is that the lifestyle drift concept is most often defined in terms of initiatives transitioning to a focus on lifestyle and changing individual choices related to health-behaviours, however, there are existing examples that relate to moving the target to healthcare and unwell populations, as well as moving to targeted programming focused on at-risk populations. Although, these second two examples are named less frequently within common definitions, they are often discussed more broadly and are argued to result from many of the same underlying mechanisms, namely neoliberalism and the biomedical model. This may be contributing to a lack of specificity in identifying case examples of lifestyle drift, as well as limiting the potential to interrupt these processes.

In addition, we would argue that lifestyle drift is a process that affects, not only policy focused on social and structural determinants, but also other initiatives that are designed to influence risk and protective factors and promote health [50]. There are several papers in our scoping review that describe cases from chronic disease foundations, not-for-profit organizations and community collaboratives [34, 36, 38] that do not have an explicit focus on policy or social determinants yet they offer examples of initiatives that are affected by lifestyle drift. It is important to recognize that lifestyle drift can have an influence at these levels as well, that may begin with a more midstream focus on risk and protective factors, yet are equally vulnerable to being drawn downstream.

Finally, the term “lifestyle” may also be too narrow in terms of the range of behaviours that appear to be implicated. The studies in our review covered a range of issues, such as self-care and parenting [30, 38], that may not fit well under the term “lifestyle choices.” In cases where there is a drift to either targeted programming or healthcare and clinical interventions, this is also not well-characterized by the term “lifestyle.” It appears that lifestyle drift may apply more broadly than lifestyle campaigns and it would be important to examine other situations where efforts are focused on educational and individualized strategies that may not have the broad benefits that they are intended to.

Given this, we suggest that the term “health intervention drift” may better reflect the underlying concept that it appears authors are trying to articulate. We would like to offer an adapted definition based on the findings of our scoping review:

(Lifestyle) drift relates to the process whereby *upstream* health promotion and primary prevention efforts are drawn *downstream* to a focus on interventions that are less likely to support intended population-level improvements in health and health equity.

In the context of this adapted definition, “(u) pstream interventions and strategies (are) those that dismantle and change the fundamental social and economic systems (structural determinants of health) that distribute the root causes of health inequities including wealth, power and opportunities. … They are about *changing the cause of the causes of health and health inequities*.” [51]. In contrast, “(d) ownstream interventions and strategies seek to address immediate needs and mitigate the negative impacts of disadvantage on health at an individual or community level through the availability of health and social services. These changes generally occur at the service or access-to-service level. Downstream strategies are about *changing the effects of the causes*.” [51].

In terms of mitigation strategies, there were two recommendations that were most commonly noted: health in all policies and inclusion of priority populations. We hope that highlighting the relative consensus on these issues helps to support increased application of these strategies. We would, however, like to highlight one caveat with respect to empowerment of priority populations. In our own work, we have noted that this approach, in itself, can inadvertently drive lifestyle drift. With respect to the implementation of the Icelandic Prevention Model, we have seen that youth advocates (both young people and adults) can be less receptive to population approaches that have demonstrated evidence, but that are implemented at a systems level. Rather, we have observed that youth advocates more commonly support interventions that focus on influencing individual youth decision-making [50], such as educational campaigns or life skills programs. In addition, in the literature reviewed within this scoping review, researchers highlight that individuals affected by health inequities are themselves susceptible to mental models of health that are influenced by the biomedical and neoliberal models [16, 24].

Therefore, we would highlight that empowerment strategies that involve working with individuals who have lived experience of particular health inequities include processes whereby individuals are meaningfully involved in learning about the related evidence on social determinants of health and informed about the project mandates and potential environmental considerations and influences, in order to make meaningful contributions based on their critical insight of first-hand experience. This direction is substantiated by the literature on critical health literacy that highlights the importance of health knowledge along with the capacity to integrate this information with lived experience and engage in meaningful dialogue related to key issues [52].

Several theoretical concepts have been used to explain the mechanisms that perpetuate health inequities and can inform future research related to lifestyle drift. For example, Bourdieu’s habitus [53], or the worldviews and dispositions developed through socialization that guide our behaviour, has been applied to explain how class divisions are perpetuated, power differentials are accepted and how these norms influence health behaviour decision-making [45]. Habitus may inform social service provider [34] and policy perspectives [16] perpetuating an emphasis on individual behavioural approaches. Sen’s Capability Approach [54] offers justification for why behavioural approaches would not be effective in circumstances where an individual does not have the necessary resources to enact healthy behaviours. The approach highlights the necessity to have the freedom and opportunity to pursue achievements that are perceived to be valuable. The capability approach has been offered as a framework to enhance health in all policy implementation by highlighting social justice and the promotion of access to opportunity across sectors [55].

The mitigation strategies described above largely operate within existing paradigms and systems. However, our findings raise the question of whether more fundamental transformations of the way we approach health equity may be beneficial. Current paradigms emphasise individual responsibility rather than structural change. It may be that incremental strategies may be more feasible in the short term, while working towards paradigm shifts for more lasting structural change.

### Strengths and limitations

This scoping review has several strengths. A librarian was consulted when developing the search strategy, and the research team includes members with experience leading scoping and systematic reviews as well as qualitative research. Combining the scoping review methodology with a thematic analysis approach brings added rigour to this study. Limitations included the possibility of missing some information about the use of the lifestyle drift concept by excluding documents that only have a passing reference to or minor focus on this concept, leading to missing some instances of application of the concept. Limiting the included documents to those in the peer reviewed literature will result in the exclusion of other uses of the lifestyle drift concept, particularly in applied settings. Future research, where time and resources permit, should expand the scope of documents examined to include the grey literature. The geographic distribution of where the authors of the papers are located was limited to North America (Canada), Europe and Oceania. Papers with first authors from the United States were also notably absent. This should be taken into account when interpreting the results. Additionally, the authors of this paper bring their own perspectives, biases and cultural orientations to the analysis and interpretation. Researchers from different contexts may have come up with different interpretations of the same information. It may be that the concept of lifestyle drift is relevant in Western contexts and may be less so in Eastern and Southern contexts. Future research should explore whether the lifestyle drift concept applies in more diverse socio-political environments. Nonetheless, this paper followed a rigorous, well-defined process to conduct a scoping review of the concept of lifestyle drift in the context of public health and will provide a foundation for development of a measurement tool to advance the understanding of this phenomenon.

For future research, we have not summarized the recommendations from the reviewed research papers as this was out of scope for the purpose of this review. However, we would like to highlight that the literature included in this review was primarily focused on narrative reviews and conceptual papers (*n* = 12) or some kind of document analysis (*n* = 14). There is a critical need for more diverse empirical research, and in particular implementation and evaluation studies that document the process of lifestyle drift. In addition, it would be useful to pursue the development of tools and/or monitoring frameworks that can be used prospectively by implementers to improve fidelity to upstream efforts and mitigate, or at least further document, the mechanics of lifestyle drift to inform future intervention design. For example, practice profiles, which help operationalize complex interventions into concrete components, can help practitioners stay on track for upstream prevention efforts [56]. Other strategies might include readiness or capacity assessments that explore contextual risk factors and potential implementation challenges related to lifestyle drift, similar to the pragmatic context assessment tool [57]. Increasing awareness of interventions designs that can support changes in living or socioeconomic conditions may also be helpful to support partners to align their work to enhance impacts on health equity [58, 59].

## Conclusion

This scoping review sheds new light on lifestyle drift by critically examining definitions, proposed causes and potential mitigating strategies. We found that characteristics of the common definitions may be limiting the uptake of information and utility of the concept. Further, we draw attention to consensus related to how lifestyle drift occurs and the ways that it can be mitigated. We hope that this work advances understanding related to implementation of upstream prevention and promotion efforts and contributes to improved outcomes related to these interventions in the future.

## Supplementary Information

Supplementary Material 1.

## Data Availability

All data and materials are be available on OSF at the following URL: https://osf.io/snfvx/.
